# Efficacy and Safety of Human Serum Albumin–Cisplatin Complex in U87MG Xenograft Mouse Models

**DOI:** 10.3390/ijms21217932

**Published:** 2020-10-26

**Authors:** Cho Rong Park, Hyo Young Kim, Myung Geun Song, Yun-Sang Lee, Hyewon Youn, June-Key Chung, Gi Jeong Cheon, Keon Wook Kang

**Affiliations:** 1 Department of Nuclear Medicine, Seoul National University Hospital, 101 Daehak-ro, Jongno-gu, Seoul 03080, Korea; sioflow304@snu.ac.kr (C.R.P.); khy4220@naver.com (H.Y.K.); wonza43@snu.ac.kr (Y.-S.L.); hwyoun@snu.ac.kr (H.Y.); jkchung@snu.ac.kr (J.-K.C.); larrycheon@gmail.com (G.J.C.); 2 Laboratory of Molecular Imaging and Therapy, Cancer Research Institute, Seoul 03080, Korea; 3 Department of Biomedical Sciences, Seoul National University Graduate School, Biomedical Science Building, Seoul 03080, Korea; 4 Biomedical Research Institute, Seoul National University Hospital, Seoul 03080, Korea; 5 Radiation Research Institute, Seoul National University College of Medicine, Seoul 03080, Korea; 6 Cancer Imaging Center, Seoul National University Hospital, Seoul 03080, Korea; 7 Institute on Aging, Seoul National University, Seoul 08826, Korea; 8 Bio-MAX Institute, Seoul National University, Seoul 08826, Korea

**Keywords:** cisplatin (CDDP), human serum albumin (HSA), cancer therapeutics, drug delivery

## Abstract

Cisplatin (cis-diamminedichloroplatinum (II), CDDP) is a chemotherapeutic drug widely used against many solid tumors. A pharmacokinetics study found that CDDP can bind to human serum albumin (HSA), which is the most abundant plasma protein in serum. HSA has the advantage of being a nanocarrier and can accumulate in tumors by passive targeting and active targeting mediated by the secreted protein acidic and rich in cysteine (SPARC). In this study, we investigated the possibility of using a CDDP–HSA complex (HSA–CDDP) as a SPARC-mediated therapeutic agent. To investigate the HSA-dependent therapeutic effect of HSA–CDDP, we used two types of U87MG glioma cells that express SPARC differently. HSA–CDDP was highly taken up in SPARC expressing cells and this uptake was enhanced with exogenous SPARC treatment in cells with low expression of SPARC. The cytotoxicity of HSA–CDDP was also higher in SPARC-expressing cells. In the tumor model, HSA–CDDP showed a similar tumor growth and survival rate to CDDP only in SPARC-expressing tumor models. The biosafety test indicated that HSA–CDDP was less nephrotoxic than CDDP, based on blood markers and histopathology examination. Our findings show that HSA–CDDP has the potential to be a novel therapeutic agent for SPARC-expressing tumors, enhancing the tumor targeting effect by HSA and reducing the nephrotoxicity of CDDP.

## 1. Introduction

Cisplatin (cis-diamminedichloroplatinum (II), CDDP) is a chemotherapeutic drug widely used against many solid tumors [1,2]. It was first described in 1845, and the antitumor potential of CDDP was first discovered in the 1960s [3]. Since its approval for clinical use in the United States in 1978, CDDP has been actively used for tumor therapy. However, CDDP is associated with significant dose-limiting toxicities, including nephrotoxicity and neurotoxicity; hence, solving this toxicity problem is important [4]. According to a pharmacokinetic study, 65–98% of CDDP administrated intravenously is bound to blood plasma proteins within one day, particularly albumin [5].

Human serum albumin (HSA) plays an important role in the transport and disposition of endogenous and exogenous ligands present in blood [6]. HSA is the most abundant plasma protein with a serum concentration of 40–45 g/L in healthy adults. It has the advantages of being a nanocarrier, water-soluble and biocompatible, and has a long half-life in blood and low toxicity [7,8]. The main reason for using HSA as a drug delivery system is its ability to accumulate in tumors by an enhanced permeability and retention (EPR) effect [9,10]. It is also known that the secreted protein acidic and rich in cysteine (SPARC), an albumin-binding protein, can sequester albumin in tumor stroma and contribute to the tumor-specific uptake of albumin [11,12]. SPARC is an albumin-binding protein highly expressed in some cancers, which functions to modulate cell–matrix interaction, proliferation, survival, and migration [13,14,15,16]. Our recent study showed that SPARC mediates the active targeting of HSA [17]. Using HSA as a CDDP carrier can also help reduce the nephrotoxicity associated with CDDP. The nephrotoxicity comes from the main excretion of CDDP, which occurs in the kidney and is due to the small size of CDDP [18,19]. By binding CDDP to HSA, HSA can inhibit the excretion of CDDP through the kidney.

CDDP bound to HSA (HSA–CDDP) is widely thought to be therapeutically inactive, but its biological effects are still controversial [20,21,22]. Some clinical trials showed increased patient survival times [23,24,25]. To utilize CDDP bound to HSA (HSA–CDDP) as a therapeutic agent, it should be examined to verify its chemotherapeutic effect. For this research, the interaction of CDDP with serum albumin was investigated at the molecular level. It is thought that five platinum atoms—His105, Met298, Met329 and Met548 and His288—can bind onto albumin molecules through the formation of coordinative bonds, and CDDP binds to His and Met side chain residues located on the albumin surface [26,27,28]. Even through this detailed structural investigation, the feasibility of HSA–CDDP as a therapeutic agent remained unsolved. Moreover, despite the high expression of SPARC in glioma, an albumin-binding protein, the feasibility of drug-carrying HSA as a therapeutic agent against glioma has not been sufficiently studied. In this study, we investigated the potential of HSA–CDDP as a therapeutic agent for a glioblastoma model and focused on the SPARC-mediated efficacy and safety.

## 2. Results

### 2.1. Characterization of HSA–CDDP

In this study, we used CDDP-bound HSA as a therapeutic drug for tumors. Based on a previous paper, we set the CDDP binding condition to HSA [29]. The molecular weight of HSA–CDDP was analyzed by the matrix-assisted laser desorption/ionization time of flight (MALDI-TOF). The molecular weight of HSA–CDDP was 67,522 ± 129 Da. The proportion of CDDP per mole of HSA was calculated from the molecular weight difference between HSA–CDDP and HSA. The average number of molecules of CDDP bound to one mole of HSA was 4.07. After we conjugated CDDP to HSA, we measured the molecular weight of HSA–CDDP using MALDI-TOF.

**Figure 1:**
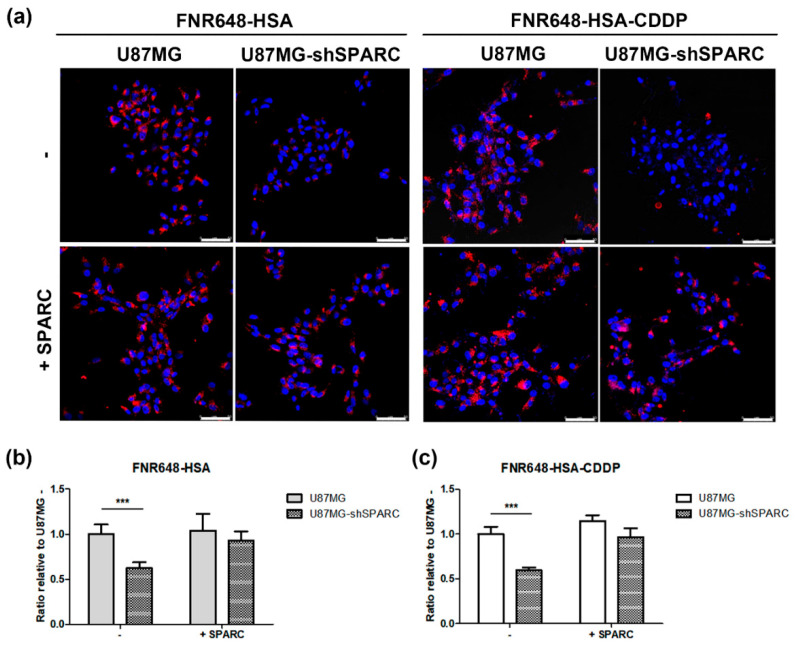
Cellular uptake of HSA–CDDP (human serum albumin–cisplatin). (**a**) Representative images of cellular uptake in cells. Exogenous secreted protein acidic and rich in cysteine (SPARC) was treated to observe SPARC dependency in HSA–CDDP uptake. Scale bars, 50 μm. (**b**) Quantification of cellular uptake of FNR648-HSA and (**c**) FNR648-HSA–CDDP in cells. The cellular uptake of each group was calculated as follows: The average signal intensity of FNR648 was divided by the number of DAPI-positive cells, which represents the number of viable cells in the image. The cellular uptake of FNR648-HSA or FNR648-HSA–CDDP in U87MG cells was considered 1 and, based on this value, we expressed each group as a ratio relative to U87MG. Data are presented as means ± SD (*n* = 5). *** *p* < 0.001.

To evaluate whether HSA–CDDP can be taken up by glioblastoma cells in a SPARC-mediated HSA-dependent manner, we performed cellular uptake imaging using confocal microscopy based on our previous paper [17,30]. Two types of U87MG cell lines were used: U87MG cells, which highly express SPARC protein; U87MG-shSPARC cells, which exhibit low expression of SPARC protein. For objective comparison, the confocal microscopic images of cells were acquired and quantified (*n* = 5 for each group). Our results showed that there is higher uptake of HSA–CDDP in U87MG cells than U87MG-shSPARC cells (Figure 1a,c, FNR648-HSA–CDDP, *p* < 0.001). To evaluate SPARC-mediated HSA–CDDP uptake in cells, exogenous SPARC protein was co-treated in cells. By co-treatment of the exogenous SPARC in cells, HSA–CDDP was highly accumulated in U87MG-shSPARC cells (Figure 1a, FNR648-HSA–CDDP in U87MG-shSPARC, and Figure 1c, +SPARC). This SPARC-dependent uptake pattern was similar to the manner of HSA uptake in cells (Figure 1a, FNR648-HSA and FNR648-HSA–CDDP). This result demonstrated that the uptake of HSA–CDDP to cells was HSA dependent.

### 2.2. Cytotoxic Effect of HSA–CDDP In Vitro

The effect of HSA–CDDP on cancer cell viability was also examined. The cytotoxicity of HSA–CDDP was studied in U87MG and U87MG-shSPAR cells using a cell counting kit-8 (CCK-8) assay. HSA–CDDP showed dose-dependent toxicity toward cells (Figure S1b, HSA–CDDP). Specifically, the toxicity of HSA–CDDP was higher with U87MG cells than U87MG-shSPARC cells (Figure S1b and Table 1; IC_50_ for HSA–CDDP in two cells). Since U87MG and U87MG-shSPARC showed similar cytotoxicity to CDDP (Figure S1a and Table 1; IC_50_ for CDDP in cells), this indicates that the cytotoxicity of HSA–CDDP to U87MG and U87MG-shSPARC is dependent on the SPARC-mediated cellular uptake of HSA–CDDP.

**Figure 2:**
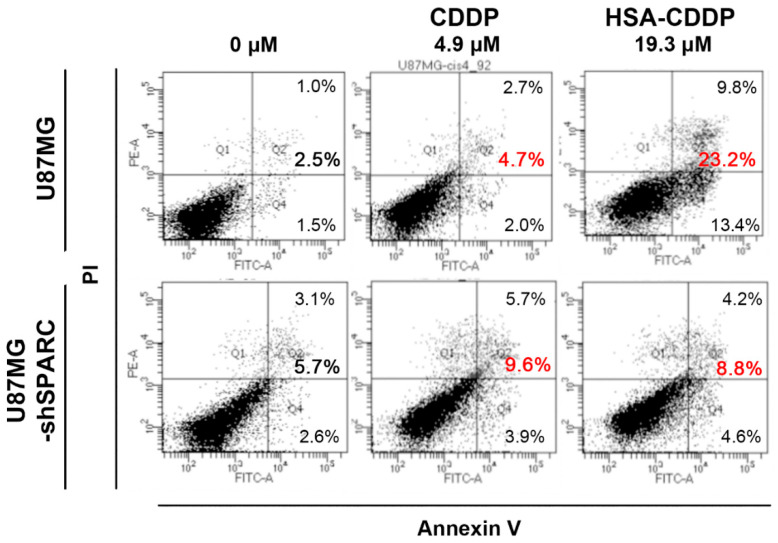
Cellular toxicity of HSA–CDDP. Apoptosis analysis of CDDP and HSA–CDDP. CDDP or HSA–CDDP was treated in cells for 72 h. Each concentration was based on the 30–40% of live cells in cck-8 results from Figure S1. The red number means total apoptosis cell percentage in each group, sum of smaller font number in each figure.

It is well known that cell apoptosis is the basic mechanism of action of CDDP [31]. Apoptosis triggered by HSA–CDDP was observed by flow cytometry analysis using Annexin V-FITC/PI co-staining (Figure 2 and Figure S2). The result showed that HSA–CDDP induced apoptosis in a dose-dependent manner (Figure S2b). We compared the percentage of apoptotic cells in the concentration of CDDP as 4.9 μM and HSA–CDDP as 19.3 μM, which showed that 30–40% of cells were alive after drug treatment in cck-8 results. U87MG exhibited a higher apoptosis rate than U87MG-shSPARC cells (Figure 2, HSA–CDDP, 19.3 μM; U87MG, 23.2%; U87MG-shSPARC, 8.8%). Comparing CDDP 4.9 μM and HSA–CDDP 19.3 in U87MG cells, HSA–CDDP exhibited a higher apoptotic cell percentage than CDDP, although they both showed similar cellular toxicity in CCK-8 (Figure 2). These results strongly indicate that the cellular toxicity of HSA–CDDP is SPARC-mediated, and HSA enhanced uptake in cells and apoptosis.

### 2.3. Antitumor Effect of HSA–CDDP In Vivo

**Figure 3:**
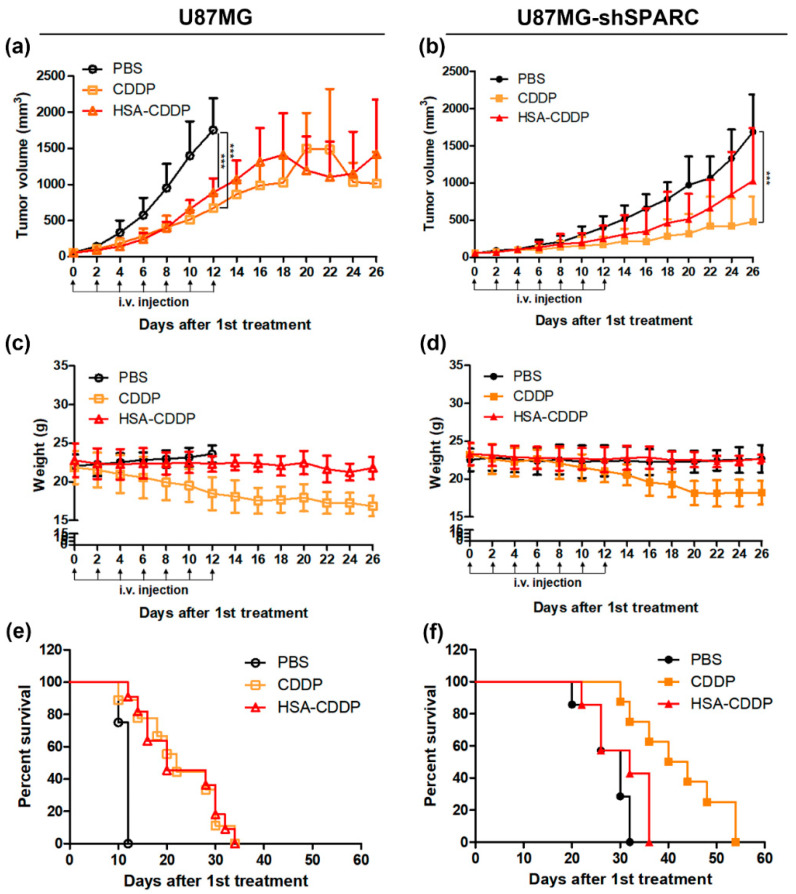
Antitumor effect of HSA–CDDP in the xenograft tumor model. Tumor volume of (**a**) U87MG and (**b**) U87MG-shSPARC. Mice weight of (**c**) U87MG and (**d**) U87MG-shSPARC in xenograft tumor mice. Kaplan–Meier survival curves for (**e**) U87MG and (**f**) U87MG-shSPARC in the tumor model. Data are presented as means ± SD. *** *p* < 0.001.

The therapeutic effect of HSA–CDDP was investigated in a xenograft tumor mice model using U87MG and U87MG-shSPARC cells. Drug administration began when tumor size reached 50 mm^3^ and tumor growth was observed until the tumor volume reached 2000 mm^3^. Mice were administered PBS, CDDP, or HSA–CDDP by intravenous injection every other day, to a total of seven times. With regard to the U87MG tumor model, 12 days after the first drug treatment, the CDDP- and HSA–CDDP-treated mice showed significantly reduced tumor growth than the PBS group (Figure 3a, *p* < 0.001). In the U87MG-shSPARC tumor model, 26 days after first drug treatment, CDDP-treated mice showed significantly reduced tumor growth than the PBS group (Figure 3b, *p* < 0.001), but HSA–CDDP-treated mice showed no difference in tumor growth to PBS. The weight of mice was significantly decreased in the CDDP-treated group (Figure 3c,d), but this did not occur with HSA–CDDP-treated mice. This means that CDDP may cause the negative effect in the in vivo system, but this effect was not seen in the HSA–CDDP treatment. For the survival rate in the U87MG tumor model, CDDP- and HSA–CDDP-treated mice exhibited a prolonged survival time than the PBS group (Figure 3e and Table 2). In the U87MG-shSPARC tumor model, HSA–CDDP treated mice showed a similar survival rate to the PBS group, whereas CDDP-treated mice showed prolonged survival (Figure 3f, Table 2). These results strongly suggest that the antitumor effect of HSA–CDDP is based on the SPARC–mediated HSA-dependent uptake in tumors and is similar to CDDP.

### 2.4. Biodistribution of HSA–CDDP In Vivo

**Figure 4:**
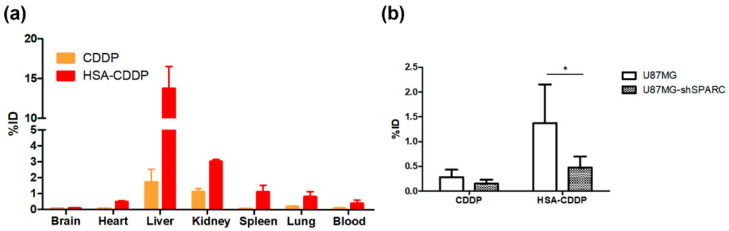
Biodistribution of HSA–CDDP in mice using ICP-MS. CDDP or HSA–CDDP was treated in the same way as therapy (every other day, seven times). (**a**) CDDP accumulation in organs and blood. (**b**) CDDP accumulation in tumors (%ID: percent injected dose). All organs and tumors were collected 72 h after the final drug treatment. Data are presented as means ± SD (*n* = 4). * *p* < 0.05.

The biodistribution of HSA–CDDP was analyzed 72 h after the last HSA–CDDP treatment as an antitumor effect (treatment administered every other day seven times, beginning 15 days after the first HSA–CDDP treatment) in normal mice and tumor-bearing mice. The amount of CDDP in each organ (brain, heart, liver, kidney, spleen, lung, and blood) was measured using inductively coupled plasma mass spectrometry (ICP-MS). HSA–CDDP showed higher blood distribution than CDDP (Figure 4a). Due to the prolonged blood distribution, all organs except the brain showed higher accumulation of CDDP in the HSA–CDDP group (Figure 4a). Particularly, HSA–CDDP was highly accumulated in the liver (Figure 4a). In the U87MG tumor accumulation of CDDP data, the amount of CDDP in the HSA–CDDP group was significantly higher than the CDDP group (Figure 4b, *p* < 0.05). In the HSA–CDDP group, U87MG tumors showed a higher accumulation of CDDP than U87MG-shSPARC tumors (Figure 4b, HSA–CDDP, *p* < 0.05). In the CDDP treatment group, there was no difference in CDDP accumulation between U87MG and U87MG-shSPARC (Figure 4b). In U87MG tumors, the HSA–CDDP group showed significantly increased CDDP accumulation than the CDDP group (Figure 4b). The result shows that HSA–CDDP is taken up by tumors in a HSA-dependent manner.

### 2.5. Biosafety of HSA–CDDP In Vivo

**Figure 5:**
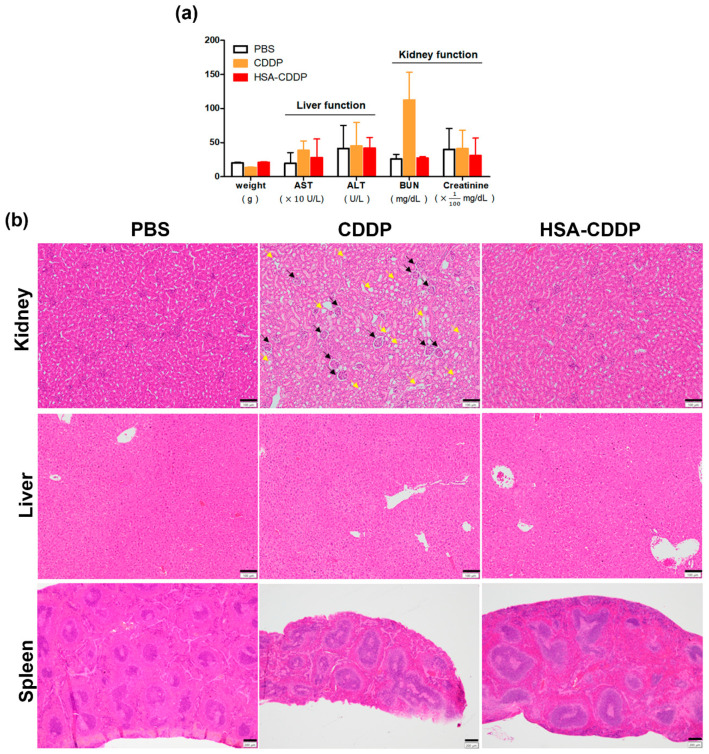
Biosafety analysis of HSA–CDDP in mice. (**a**) Body weight and serum concertation of aspartate transaminase (AST), alanine aminotransferase (ALT), blood urea nitrogen (BUN), and creatinine. (**b**) Organ staining images using hematoxylin–eosin (H&E) in the kidney, liver, and spleen section. Tubular degeneration in the kidney (black arrows) and epithelial vacuolization (yellow arrows) were observed in the kidney of mice treated with CDDP. Scale bars, 100 μm for kidney and liver, 200 μm for spleen.

In the antitumor effect result, mice treated with CCDP showed significant weight loss compared to the group treated with PBS. This result shows that there can be negative effects of the CDDP in vivo system, and this needs to be verified. It is well known that the major dose-limiting side effect of CDDP is nephrotoxicity [4]. In biodistribution studies, HSA–CDDP was highly accumulated in the liver. We assessed the toxicity of HSA–CDDP in the mice by monitoring the blood marker of liver, kidney function, and body weight 72 h after the final HSA–CDDP treatment as an antitumor effect plan (every other day, seven times, 15 days after the first HSA–CDDP treatment). The CDDP treated group showed significant body weight loss compared to the PBS and HSA–CDDP groups, similar to the antitumor effect result for weight (Figure 5a). In terms of liver function, aspartate transaminase (AST) and alanine aminotransferase (ALT) showed no significant difference among groups (Figure 5a). For kidney function, blood urea nitrogen (BUN) increased in the CDDP group (Figure 5a). In the hematoxylin–eosin (H&E) staining images, CDDP treated kidney tissue showed tubular degeneration (Figure 5b, black arrows) and extensive epithelial vacuolization (Figure 5b, yellow arrows). In the terminal deoxynucleotidyl transferase (TUNEL) assay, TUNEL positive cells were only identified in the kidney of CDDP-treated mice (Figure S3). From this result, it was confirmed that HSA–CDDP reduced the nephrotoxicity of CDDP in vivo.

## 3. Discussion

The current standard therapy for glioma is surgical resection. However, it is difficult to remove all gliomas completely because they are occasionally located in the functional brain area [32]. There are other issues related to using CDDP for glioma therapy, as a limited amount of any given platinum drug dose was delivered due to the blood–brain barrier (BBB) in the brain [33,34,35]. Using the HSA–CDDP complex as a therapeutic agent can be a solution to eliminate the remnant glioma in the brain.

The interaction of different platinum-based drugs with albumin has received much attention in the last 30 years [36,37]. HSA is a famous nanocarrier that can prolong the blood circulation time and enhance tumor accumulation by the EPR effect and SPARC-mediated active targeting [9,10,17]. In this study, we used HSA–CDDP as a therapeutic complex, using HSA as a nanocarrier.

The cellular uptake of HSA–CDDP was SPARC-dependent. In vitro toxicity results showed that SPARC-expressing U87MG cells exhibited higher cellular toxicity to HSA–CDDP. The antitumor effect of HSA–CDDP also showed a SPARC dependent HSA-mediated therapeutic effect in U87MG, which was similar to that of CDDP. The tumor accumulation of CDDP was significantly higher in HSA–CDDP treated U87MG tumors.

The cellular cytotoxicity data revealed that the cytotoxicity of HSA–CDDP was lower than CDDP alone. However, CDDP and HSA–CDDP showed a similar antitumor effect in vivo. There are two possible explanations for this: First, it can be postulated from the characteristics of SPARC, which is a secreted form of protein from tumor cells. SPARC is known to facilitate albumin accumulation in tumor stroma [12,38]. As the in vitro environment is somewhat different to the in vivo tumor environment, SPARC-mediated HSA accumulation may not be as dominant in vitro. Another reason may be due to the enhanced blood circulation time of HSA–CDDP by the nanocarrier of HSA. The half-life of CDDP in plasma is 0.27 h [39].

SPARC is highly expressed in many tumors, such as glioma, melanoma, and breast cancer [40,41]. In this study, we used glioma cell line U87MG, which exhibits the highest expression of SPARC in the cell line. To broaden the applicability of HSA–CDDP as a tumor targeting therapeutic effect, studies using other SPARC expressing tumors are also required.

CDDP binding to HSA is known to be irreversible [42,43,44]. We showed the irreversible binding of CDDP to HSA through the serum stability test, where no free CDDP was detected from HSA–CDDP incubation with serum (Figure S4a). Due to the irreversible binding of CDDP to HSA, it can be postulated that the distribution of HSA–CDDP can be similar to that of HSA. From the single photon emission computed tomography (SPECT) tests on ^177^Lu labeled HSA and HSA–CDDP distribution imaging in mice, HSA–CDDP showed a similar distribution and blood circulation to HSA (Figure S4b). Due to the HSA-dependent pharmacokinetics of HSA–CDDP and enhanced blood circulation in vivo, HSA–CDDP was highly accumulated in tumors and showed a similar therapeutic effect to that of HSA–CDDP in U87MG tumors.

The mechanism of action of CDDP is based on the aquation of CDDP in cytoplasm because of the reduced cytoplasmic concentration of chloride ions [45,46]. This aquated CDDP can bind with nuclear DNA and induce a DNA damage response [47]. Aquated CDDP can also induce the accumulation of reactive oxygen species (ROS) or can physically interact with cytoplasmic nucleophiles such as mitochondrial DNA and proteins [48,49,50,51]. As a result of this activity, cells die via mitochondrial apoptosis [52]. During CDDP binding to HSA, CDDP loses chloride ions [28]. In light of the therapeutic effect of CDDP, it is unfavorable to use HSA–CDDP as an anticancer agent. This could be the reason for the similar therapeutic effect observed for HSA–CDDP and CDDP in the in vivo tumor models, despite the enhanced tumor accumulation of HSA–CDDP.

Without free CDDP release from HSA–CDDP, HSA–CDDP showed an antitumor effect in vitro and in vivo. It can be postulated that HSA–CDDP can be taken up to cells by endocytosis and then degradation of HSA–CDDP could release free CDDP from HSA [53,54]. The detailed anticancer mechanism of HSA–CDDP at the cellular level is unknown. Further studies are needed to unravel the underlying mechanism of action of HSA–CDDP as a therapeutic agent at the cellular level.

CDDP induces nephrotoxicity. CDDP is primarily excreted by the kidney where it is accumulated during the excretion process, preferentially in the S3 segment of the renal proximal tubules [55]. The accumulation of CDDP in proximal tubular cells is also mediated by the membrane transporters copper transporter-1 and organic cation transporter-2 (OCT2), which are mainly expressed in the basolateral membrane of renal proximal tubular cells [56,57]. A biosafety test of the blood marker and structural observations indicated that only the CDDP treated group showed increased kidney function blood marker (BUN) and structural malformation of the kidney. As a result of HSA being bound to CDDP, kidney accumulation of CDDP mediated by excretion and OCT2 can be reduced.

In the biodistribution data, HSA–CDDP showed a higher accumulation in all normal organs than CDDP. We observed the biosafety of HSA–CDDP in mice by monitoring the blood marker and body weight 72 h after the last HSA–CDDP treatment. This biosafety marker and histological imaging showed no liver toxicity, but this result only represents short-term organ toxicity. In the liver, HSA–CDDP degradation can occur and this can cause the release of CDDP from the liver and induce late organ toxicity. To insist on the low toxicity effect of HSA–CDDP, long-term organ toxicity studies are needed.

The possibility that a CDDP adduct of serum albumin can be used as a therapeutic agent is still highly controversial and has not been well investigated [58,59]. In this study, we showed that the HSA–CDDP complex can be used as a therapeutic agent and achieves a similar therapeutic effect to CDDP. Using HSA–CDDP as a therapeutic agent has some advantages: HSA–CDDP has a higher molecular size than CDDP, which can enhance the blood circulation time of CDDP and reduce the nephrotoxicity caused by the rapid excretion of CDDP through the kidney. In this study, we showed that this advantage worked well in an in vivo model, reducing nephrotoxicity. HSA-mediated tumor targeting can enhance the accumulation of CDDP in tumors, through the EPR effect or active tumor targeting. In this study, we showed that HSA–CDDP can target tumors in a SPARC-mediated HSA-dependent manner.

## 4. Materials and Methods

### 4.1. Cell Lines

Human glioma cells (U87MG) were obtained from American Type Culture Collection (ATCC; Manassas, VA, USA). Low SPARC-expressing U87MG cells, U87MG-shSPARC, were established from a previous study [17]. Cells were grown in Minimum Essential Medium (MEM; Gibco, Grand Island, NY, USA) supplemented with 10% heat-inactivated fetal bovine serum (FBS; Gibco, Grand Island, NY, USA) and 1% antibiotics containing penicillin/streptomycin (Invitrogen, Grand Island, NY, USA). All cells were maintained at 37 °C in a humidified atmosphere with 5% CO_2_.

### 4.2. Preparation of CDDP Conjugated HSA

Human serum albumin (MP biomedicals, Irvine, CA, USA) was dissolved in PBS as 22 mg/mL concentration. CDDP (Sigma-Aldrich, St. Louis, MO, USA) was dissolved in PBS as a 1 mg/mL concentration. HSA and CDDP solutions were added to a new bottle at a molar ratio of 1:10 (1:1 volume ratio) and stirred for 24 h at 37 °C. To remove unconjugated CDDP and concentrate HSA–CDDP, centrifugal filtration was conducted using an Amicon Ultra centrifugal filter unit (nominal molecular weight limit 30 kDa; Millipore, Burlington, MA, USA). The HSA–CDDP concentration was measured with a bicinchoninic acid (BCA) protein assay kit (Pierce Endogen, Rockford, IL, USA), and the molecular weight was analyzed by matrix-assisted laser desorption/ionization-time of flight (MALDI-TOF) using the TOF-TOF 5800 System (AB SCIEX, Framingham, MA, USA) to check the amount of CDDP per HSA in HSA–CDDP.

### 4.3. Conjugation of Fluorescence Dye to HSA and HSA–CDDP

We conjugated HSA with FNR648 fluorescence dye following the procedure described in our previous publication [60]. For FNR648 dye labeling to HSA–CDDP, we used the same method as that used for HSA. First, HSA–CDDP was modified using dibenzocyclooctyne (DBCO)-NHS. DBCO–HSA–CDDP was reacted with FRN648 dye at a molar ratio of 1:1 for 30 min at 37 °C. Fluorescence labeled HSA–CDDP was purified using PD-10 columns (GE Healthcare, Buckinghamshire, UK) and eluted with PBS.

### 4.4. Confocal Microscopy Imaging for Cellular Uptake of FNR648-HSA–CDDP

Cellular uptake of fluorescence labeled HSA or HSA–CDDP followed the same procedure described in our previous publication [17,30]. We incubated cells with FNR648-HSA or FNR648-HSA–CDDP for 2 h at 37 °C. In the exogenous SPARC treatment group, human SPARC (5 μg/mL) and each compound (FNR648-HSA or FNR648-HSA–CDDP) were co-incubated for 2 h at 37 °C. Five randomized images were acquired from all tests to quantify FNR648-HSA or FNR648-HSA–CDDP uptake by the cells. In each group, the average signal intensity of FNR648-HSA or FNR648-HSA–CDDP was divided by the number of DAPI-positive cells, which represented the number of viable cells in the image. This ratio was considered as cellular uptake of each group and used to quantify cellular uptake of each material.

### 4.5. Cellular Cytotoxicity Studies

U87MG and U87MG-shSPARC cells were added to 96-well plates (4000 cells/well). After overnight incubation, the cells were incubated with CDDP, HSA–CDDP at different concentrations for 72 h. Cell viability was analyzed using a Cell Counting Kit-8 (CCK-8, Dojindo Molecular Technologies, Tokyo, Japan) assay according to the manufacturer’s protocols.

### 4.6. Cell Apoptosis Study

U87MG and U87MG-shSPARC cells were placed into 6-well plates (1.2 × 10^5^ cells/well). After overnight incubation, cells were treated with CDDP, HSA–CDDP for 72 h. Cells were then harvested and co-stained with PI and Annexin V using an Annexin V-FITC apoptosis detection kit (BD, San Jose, CA, USA). The apoptosis of cells was analyzed using flow cytometry.

### 4.7. Animal Xenografts Tumor Model and Anti-Tumor Effect In Vivo

All animal studies were performed under approval from the Seoul National University Institutional Animal Care and Use Committee (IACUC No. 18-0231, 1 November 2018). BALB/c nude mice (5-week-old, male) were purchased from Orient Bio Inc. (Seongnam, Korea). U87MG or U87MG-shSPARC cells (2 × 10^6^ cells/site) were injected subcutaneously into the right lower flanks. When tumor volume approached 50 mm^3^, mice were randomly divided into 3 groups (U87MG: *n* = 12 for PBS group, *n* = 9 for CDDP group and *n* = 11 for HSA–CDDP group. U87MG-shSPARC: *n* = 7 for PBS group, *n* = 6 for CDDP group and *n* = 9 for HSA–CDDP group). Mice were IV administrated (7 times, every other day) with PBS, CDDP, or HSA–CDDP. The drug dose of CDDP was 3 mg/kg and the dose for HSA–CDDP was the equivalent amount of CDDP (3 mg/kg CDDP from HSA–CDDP). The tumor size and body weight of each mouse were recorded every other day. Tumor volume was calculated using the equation V = 0.5 × L × W^2^, where L represents tumor length and W represents tumor width. To evaluate the survival rate of CDDP and HSA–CDDP in the tumor model, mice were monitored and euthanized following the humane endpoints guideline (specifically, rapid weight loss of 15–20% within a few days or tumor volume is larger than 2000 mm^3^).

### 4.8. Biodistribution of CDDP Using ICP-MS

Normal mice were divided into 2 groups (*n* = 4 for each group) and IV administrated with CDDP or HSA–CDDP at the same dose and time schedule as the anti-tumor effect examination (3 mg/kg CDDP and equivalent dose of HSA–CDDP as CDDP every other day, seven times). At 72 h after the final IV administration (15 days after first IV administration), mice were euthanized and organs (brain, heart, liver, kidney, spleen, and lung) and blood were acquired and weighed. For tumor CDDP distribution, U87MG or U87MG-shSPARC cells (2 × 10^6^ cells/site) were injected subcutaneously into the right lower flanks. When tumor volume approached 50 mm^3^, mice were randomly divided into 2 groups (*n* = 5 for each group) and IV administrated CDDP or HSA–CDDP (every other day, seven times). At 72 h after the last IV administration (15 days after first administration), mice were euthanized, and the tumor was acquired. All samples (organs, blood, and tumors) were lyophilized and we analyzed the amount of CDDP using the ICP-MS (NexION 350; Perkin-Elmer, Waltham, MA, USA) installed at the National Center for Inter-university Research Facilities (NCIRF) at Seoul National University.

### 4.9. Hematology Analysis

Mice were treated with PBS, CDDP, or HSA–CDDP (*n* = 4 for each group) as the therapy treatment (every other day, seven times) and murine blood samples were acquired at 72 h after the final IV administration (15 days after first treatment). After centrifugation at 4 °C for 20 min, the plasma was collected for blood biochemical analysis. The concentrations of AST, ALT, BUN, and creatinine were analyzed at DKKorea (Seoul, Korea).

### 4.10. Histopathology Examination

The kidney, liver, and spleen of the drug-treated mice were acquired and fixed with 4% paraformaldehyde. Tissues were embedded in paraffin and stained with hematoxylin and eosin (H&E). H&E staining images were acquired using an optical microscope (Olympus BX43, Tokyo, Japan).

### 4.11. Serum Stability of HSA–CDDP

The CDDP stability of HSA–CDDP in serum was examined following method in another publication [61]. Serum albumin/HSA–CDDP solution were incubated at 37 °C and free CDDP in serum was measured at 0.5, 1, 2, 4, 8, 16, 24, 48, 72 and 120 h after incubation. To obtain free CDDP from serum, an Amicon Ultra centrifugal filter unit (nominal molecular weight limit 30 kDa; Millipore, Burlington, MA, USA) was used. The CDDP concentration measured in the supernatant (bottom of the tube, smaller than 30 KDa) corresponded to the free CDDP level. The concentration of CDDP in the supernatant was analyzed using the ICP-MS (NexION 350; PerkinElmer, Waltham, MA, USA) installed at the National Center for Inter-university Research Facilities (NCIRF) at Seoul National University.

### 4.12. SPECT Imaging

Small animal SPECT imaging of tumor-bearing mice was performed using Nano SPECT/CT plus (Mediso medical imaging system, Budapest, Hungary). Mice were injected with 18.5 MBq of ^177^Lu-HSA or ^177^Lu-HSA–CDDP via the tail vein. SPECT images were acquired at 10 min, 4 h, 24 h, 48 h and 72 h after injection. To acquire SPECT images, mice were anesthetized with 2% isoflurane and placed in the prone position. SPECT scans were acquired at 30 s per frame and 40 projections (frames) at an 18° angular step. The energy peaks of ^177^Lu were set to 56.1 keV ± 10%, 112.9 keV ± 10%, and 208.4 keV ± 10%. Reconstructed data from SPECT were visualized using InVivoScope (Bioscan, Washington, DC, USA).

### 4.13. Immunohistochemistry and TUNEL Assay

The tumor and kidney were fixed with 4% paraformaldehyde and embedded in paraffin, which was further cut into 4 μm sections. To evaluate cellular apoptosis in kidney, a kidney section was stained using a TUNEL assay kit-HRP-DAB (ab206386; abcam, Cambridge, UK) according to the manufacturer’s protocols.

### 4.14. Statistical Analysis

All statistical analyses were performed using GraphPad Prism. Student’s t-test was used to determine the statistical significance of cellular uptake of HSA–CDDP, the antitumor effect of HSA–CDDP in the xenograft tumor model, and the biodistribution of HSA–CDDP in mice. *P* values below 0.05 were considered statistically significant.

## 5. Conclusions

In this study, the HSA–CDDP conjugate examined exhibited SPARC-dependent HSA-mediated tumor accumulation in a U87MG xenograft mouse model. The therapeutic effect of HSA–CDDP was similar to that of CDDP, but nephrotoxicity—the major dose-limiting effect of CDDP—was reduced. In conclusion, we showed the feasibility of CDDP-carrying HSA as a therapeutic agent against glioma.

## Figures and Tables

**Table 1:** IC_50_ values for CDDP and HSA–CDDP in cells. Data are presented as means ± SD (*n* = 5).

IC50 (μM)	U87MG	U87MG-shSPARC
CDDP	2.545 ± 0.1345	2.646 ± 0.1302
HSA–CDDP	11.49 ± 0.1726	≈59.3

**Table 2:** Median survival in days of U87MG and U87MG-shSPARC tumor xenograft model.

Median Survival (Days)	PBS	CDDP	HSA–CDDP
U87MG	12	22	20
U87MG-shSPARC	30	42	32

## Supplementary Materials

Supplementary Materials can be found at https://www.mdpi.com/1422-0067/21/21/7932/s1.

## Abbreviations

CDDP	Cis-diamminedichloroplatinum (II)
HSA	Human serum albumin
SPARC	Secreted protein acidic and rich in cysteine
EPR	Enhanced permeability and retention
MALDI-TOF	Matrix-assisted laser desorption/ionization-time of flight
CCK-8	Cell counting kit-8
PBS	Phosphate buffered saline
ICP-MS	Inductively coupled plasma-mass spectrometry
%ID	Percent injected dose
AST	Aspartate transaminase
ALT	Alanine aminotransferase
BUN	Blood urea nitrogen
H&E	Hematoxylin-eosin
TUNEL	Terminal deoxynucleotidyl transferase
SPECT	Single photon emission computed tomography
OCT2	Organic cation transporter-2
ROS	Reactive oxygen species
BBB	Blood–brain barrier
MEM	Minimum essential medium
FBS	Fetal bovine serum
BCA	Bicinchoninic acid
DBCO	Dibenzocyclooctyne
