# Supplementary material for: Efficacy and Safety of Human Serum Albumin–Cisplatin Complex in U87MG Xenograft Mouse Models
Source: Int J Mol Sci. 2020 Oct 26;21(21):7932. doi: 10.3390/ijms21217932 (PMC7663476; doi:10.3390/ijms21217932)
Supplement: Supplementary file 1 [file ijms-21-07932-s001.pdf]

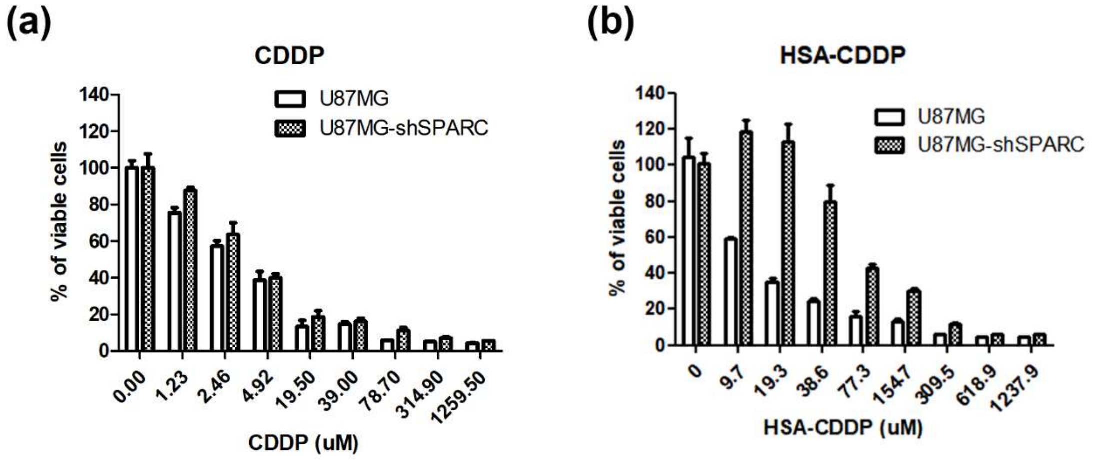

Figure S1. Cellular toxicity of CDDP and HSA-CDDP in U87MG and U87MG-shSPARC cells. (a) Cytotoxicity of CDDP in U87MG and U87MG-shSPARC cells. (b) Cytotoxicity of HSA-CDDP in U87MG and U87MG-shSPARC cells (n = 5 for each concentration). Each compound was treated 72 h.

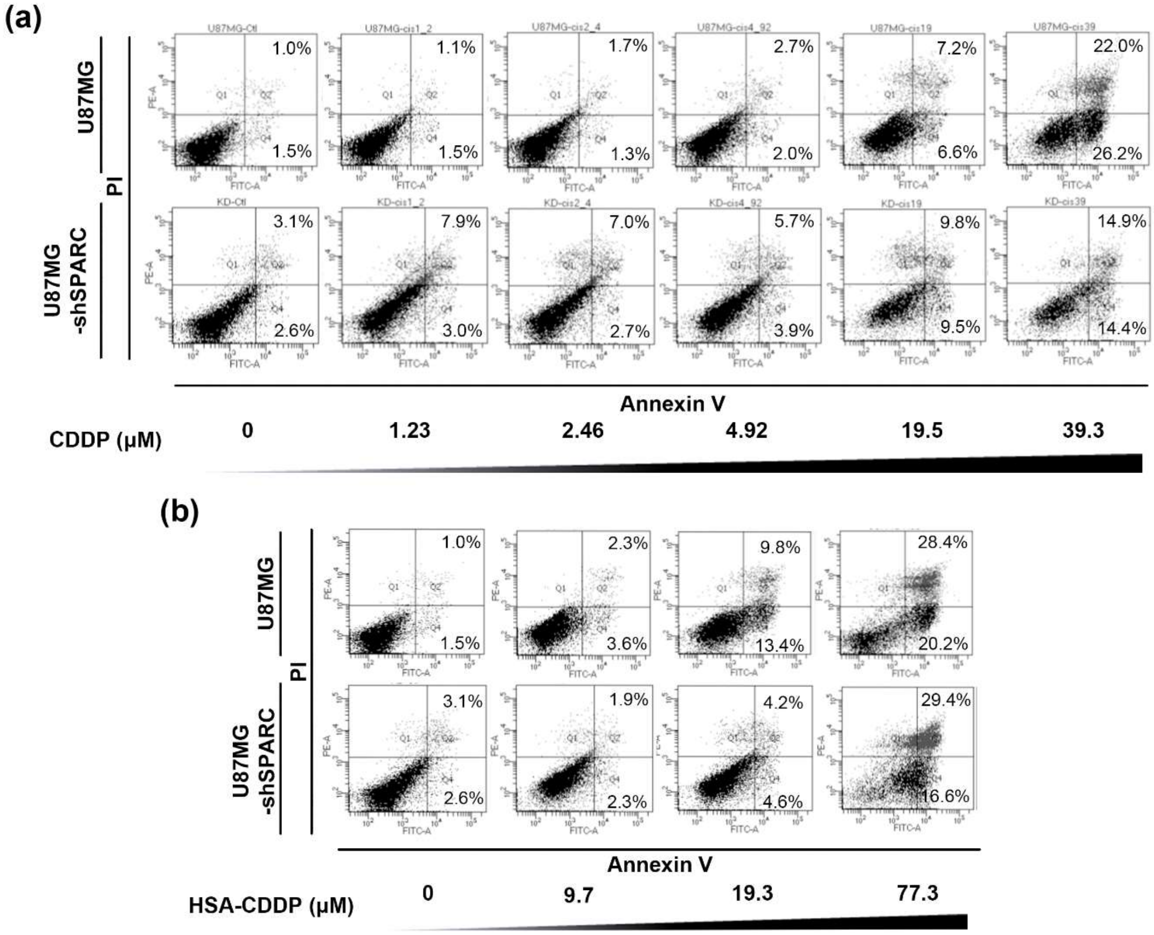

Figure S2. Apoptosis analysis of CDDP and HSA-CDDP in U87MG and U87MG-shSPARC cells. (a) CDDP and (b) HSA-CDDP were treated in cells 72 h for each concentration.

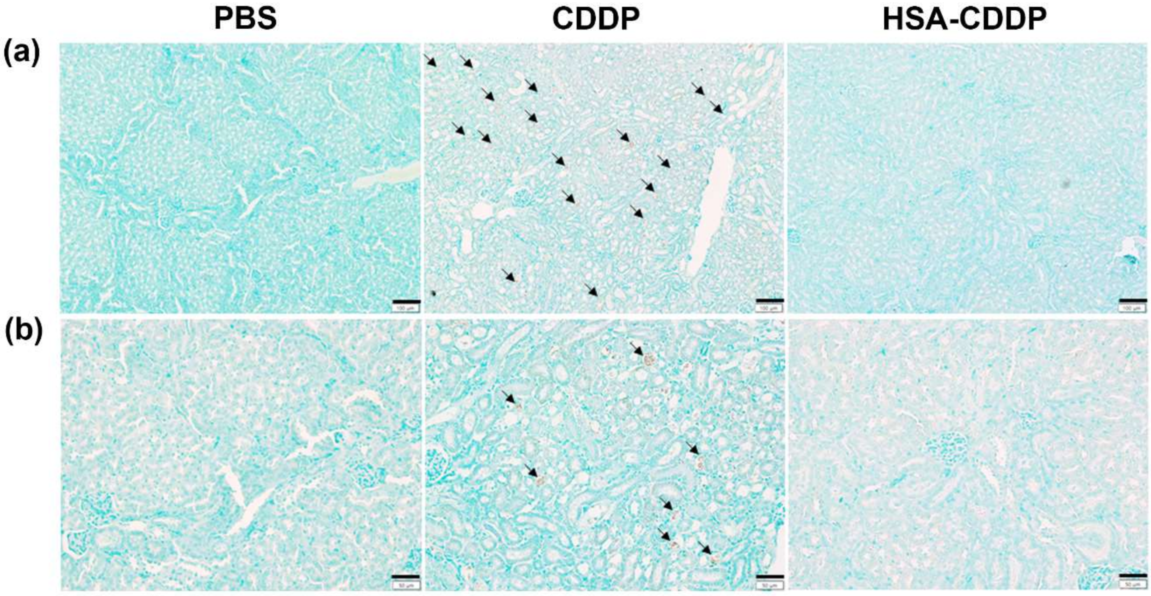

Figure S3. TUNEL assay images in the kidney tissue. (a) 200x images. Scale bars, 100 μm. (b) 400x images. Scale bars, 50 μm. Black arrow represents TUNEL positive area.

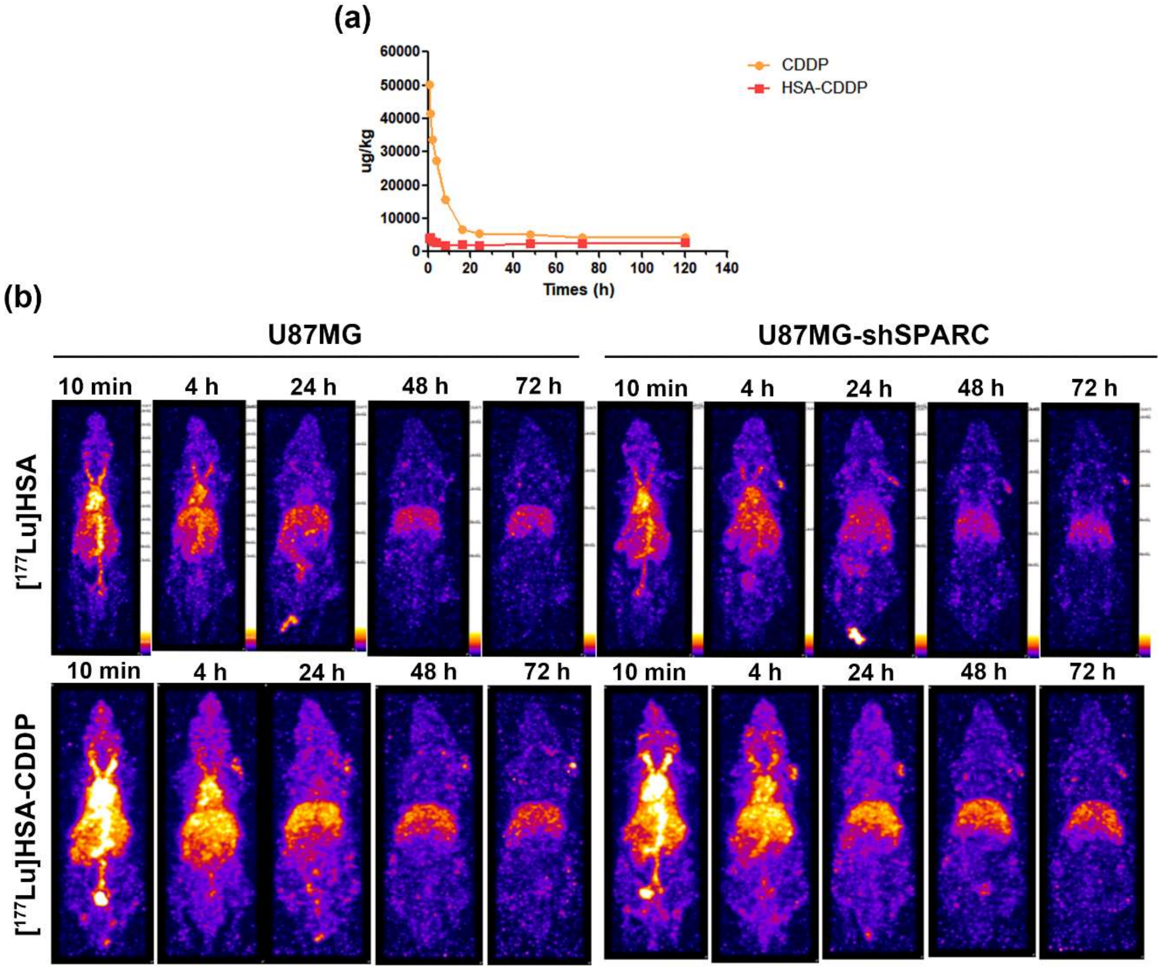

Figure S4. Serum stability of HSA-CDDP and biodistribution imaging. (a) Serum stability of HSA-CDDP *in vitro*. Each time point, samples were centrifugal filter to obtain CDDP and the amount of CDDP in sample was measured using ICP-MS. (b) SPECT images of [^{177}Lu] labeled HSA-CDDP in tumor xenograft models.
